# Representation of cardiovascular magnetic resonance in the AHA / ACC guidelines

**DOI:** 10.1186/s12968-017-0385-z

**Published:** 2017-09-25

**Authors:** Florian von Knobelsdorff-Brenkenhoff, Guenter Pilz, Jeanette Schulz-Menger

**Affiliations:** 1 Department of Cardiology, Clinic Agatharied, Ludwig-Maximilians-University Munich, Norbert-Kerkel-Platz, 83734 Hausham, Germany; 2 Charité – Universitätsmedizin Berlin, corporate member of Freie Universität Berlin, Humboldt-Universität zu Berlin, and Berlin Institute of Health, DZHK (German Centre for Cardiovascular Research), partner site Berlin, Berlin, Germany; 3 Working Group Cardiovascular Magnetic Resonance, Experimental and Clinical Research Center, a joint cooperation between the Charité Medical Faculty and the Max-Delbrueck Center for Molecular Medicine and HELIOS Klinikum Berlin Buch, Department of Cardiology and Nephrology, Berlin, Germany

**Keywords:** Cardiac magnetic resonance, Guideline, Cardiology, Reimbursement

## Abstract

**Background:**

Whereas evidence supporting the diagnostic value of cardiovascular magnetic resonance (CMR) has increased, there exists significant worldwide variability in the clinical utilization of CMR. A recent study demonstrated that CMR is represented in the majority of European Society for Cardiology (ESC) guidelines, with a large number of specific recommendations in particular regarding coronary artery disease. To further investigate the gap between the evidence and clinical use of CMR, this study analyzed the role of CMR in the guidelines of the American College of Cardiology (ACC) and American Heart Association (AHA).

**Methods:**

Twenty-four AHA/ACC original guidelines, updates and new editions, published between 2006 and 2017, were screened for the terms “magnetic”, “MRI”, “CMR”, “MR” and “imaging”. Non-cardiovascular MR examinations were excluded. All CMR-related paragraphs and specific recommendations for CMR including the level of evidence (A, B, C) and the class of recommendation (I, IIa, IIb, III) were extracted.

**Results:**

Twelve of the 24 guidelines (50.0%) contain specific recommendations regarding CMR. Four guidelines (16.7%) mention CMR in the text only, and 8 (33.3%) do not mention CMR. The 12 guidelines with recommendations for CMR contain in total 65 specific recommendations (31 class-I, 23 class-IIa, 6 class-IIb, 5 class-III). Most recommendations have evidence level C (44/65; 67.7%), followed by level B (21/65; 32.3%). There are no level A recommendations. 22/65 recommendations refer to vascular imaging, 17 to congenital heart disease, 8 to cardiomyopathies, 8 to myocardial stress testing, 5 to left and right ventricular function, 3 to viability, and 2 to valvular heart disease.

**Conclusions:**

CMR is represented in two thirds of the AHA/ACC guidelines, which contain a number of specific recommendations for the use of CMR. In a simplified comparison with the ESC guidelines, CMR is less represented in the AHA/ACC guidelines in particular in the field of coronary artery disease.

## Background

The body of evidence supporting the beneficial utilization of cardiovascular magnetic resonance (CMR) has grown significantly over the last decade [1, 2]. A recent analysis exhibited that CMR is already incorporated into 88% of the guidelines published by the European Society of Cardiology, in many as specific recommendations, and in most at least by mention in the text passages [3]. Hence, CMR commonly plays a role in evidence based diagnostic and therapeutic pathways, and can even be considered mandatory in a number of clinical scenarios. However, in the experience of the authors, the integration of CMR into clinical medicine appears to still be limited relative to the growing evidence supporting its benefits. This discrepancy may be attributed to a number of factors, such as limited access to scanners equipped for CMR, lack of people with the necessary skills to run and interpret a CMR study, relatively high costs, competing diagnostic modalities, and inadequate reimbursement. The guidelines published by the American Heart Association (AHA) and the American College of Cardiology (ACC) are often used as the basis for clinical decision making and therefore can have a high impact on utilization of technology such as CMR. This analysis systematically summarizes the representation of CMR in the AHA/ACC guidelines to stimulate the discussion about future needs for training, distribution of equipment, and reimbursement of CMR worldwide.

## Methods

All AHA/ACC guidelines published between 2006 and June 2017 and listed on the AHA and ACC websites were collected (Table 1). If more than one guideline for the same topic was published during this period, the most recent was included in the analysis. If a guideline was updated, both the full guideline and the update were analyzed in combination. The documents were screened for the terms “magnetic”, “MRI”, “CMR”, “MR” and “imaging”. MRI in the context of non-cardiovascular examinations, such as brain MRI, was not included. The main conclusions were extracted, and if available, the class of recommendation and the level of evidence were given. The class of recommendation (i.e., the strength of the recommendation) encompasses the anticipated magnitude and certainty of benefit in proportion to risk (Table 2). The level of evidence rates evidence on the basis of the type, quality, quantity, and consistency of data from clinical trials and other reports (Table 3) [4]. Whereas recent guidelines separate levels B and C into sublevels, earlier guidelines did not. In this analysis, the level as provided in each guideline is given. The number in parenthesis behind the citation provides the page number of the full text guideline. If a recommendation refers to “imaging” in general, it was registered only if the context included CMR. This analysis was performed twice for every guideline to assure that no relevant information was missed. The absolute number of recommendations was then summarized. The guidelines are listed in chronologic order beginning with the most recent. Only USA-guidelines published by the AHA and ACC were included; AHA/ACC position statements, and guidelines published by other organizations, were not included to guarantee consistency.

**Table 1:** List of AHA/ACC guidelines used for the analysis. +++ = guideline contains specific recommendations regarding the use of CMR; ++ = guideline mentions scenarios in which CMR may be used, but without giving any specific recommendation; + = guideline does not mention CMR at all

Nr.	Title	Year	Role of CMR	I	IIa	IIb	III
1	▪ ACC/AHA/HFSA Focused Update of the 2013 ACCF/AHA Guideline for the Management of Heart Failure [5] ▪ ACC/AHA/HFSA Focused Update on New Pharmacological Therapy for Heart Failure: An Update of the 2013 ACCF/AHA Guideline for the Management of Heart Failure [6] ▪ ACCF/AHA Guideline for the Management of Heart Failure [22]	2017 2016 2013	+++	0	2	2	0
2	▪ AHA/ACC Focused Update of the 2014 AHA/ACC Guideline for the Management of Patients With Valvular Heart Disease [7] ▪ AHA/ACC Guideline for the Management of Patients With Valvular Heart Disease [23]	2017 2014	+++	5	0	1	0
3	▪ ACC/AHA/HRS Guideline for Evaluation and Management of Patients With Syncope [24]	2017	+++	0	1	1	0
4	▪ AHA/ACC Guideline on the Management of Patients With Lower Extremity Peripheral Artery Disease [4]	2016	+++	1	0	0	1
5	▪ ACC/AHA/HRS Guideline for the Management of Adult Patients With Supraventricular Tachycardia [25]	2015	+				
6	▪ ACC/AHA/SCAI Focused Update on Primary Percutaneous Coronary Intervention for Patients With ST-Elevation Myocardial Infarction: An Update of the 2011 ACCF/AHA/SCAI Guideline for Percutaneous Coronary Intervention and the 2013 ACCF/AHA Guideline for the Management of ST-Elevation Myocardial Infarction [10] ▪ ACCF/AHA Guideline for the Management of ST-Elevation Myocardial Infarction [11]	2015 2013	+				
7	▪ ACCF/AHA/SCAI Guideline for Percutaneous Coronary Intervention [12]	2011	++				
8	▪ ACC/AHA Guideline on Perioperative Cardiovascular Evaluation and Management of Patients Undergoing Noncardiac Surgery [15] ▪ ACC/AHA Guidelines on Perioperative Cardiovascular Evaluation and Care for Noncardiac Surgery [16]	2014 2007	++				
9	▪ ACC/AHA/AATS/PCNA/SCAI/STS Focused Update of the Guideline for the Diagnosis and Management of Patients With Stable Ischemic Heart Disease [8] ▪ ACCF/AHA/ACP/AATS/PCNA/SCAI/STS Guideline for the Diagnosis and Management of Patients With Stable Ischemic Heart Disease [17]	2014 2012	+++	1	4	0	3
10	▪ AHA/ACC Guideline for the Management of Patients With Non–ST-Elevation Acute Coronary Syndromes [26]	2014	+++	1	0	0	0
11	▪ AHA/ACC/HRS Guideline for the Management of Patients With Atrial Fibrillation [27]	2014	++				
12	▪ AHA/ACC/TOS Guideline for the Management of Overweight and Obesity in Adults [28]	2013	+				
13	▪ AHA/ACC Guideline on Lifestyle Management to Reduce Cardiovascular Risk [29]	2013	+				
14	▪ ACC/AHA Guideline on the Treatment of Blood Cholesterol to Reduce Atherosclerotic Cardiovascular Risk in Adults [30]	2013	+				
15	▪ ACC/AHA Guideline on the Assessment of Cardiovascular Risk [31]	2013	+				
16	▪ ACCF/AHA/HRS Focused Update Incorporated Into the ACCF/AHA/HRS 2008 Guidelines for Device-Based Therapy of Cardiac Rhythm Abnormalities [9] ▪ ACC/AHA/HRS Guidelines for Device-Based Therapy of Cardiac Rhythm Abnormalities [32]	2012 2008	++				
17	▪ ACCF/AHA Guideline for the Diagnosis and Treatment of Hypertrophic Cardiomyopathy [33]	2011	+++	2	1	3	0
18	▪ ACCF/AHA Guideline for Coronary Artery Bypass Graft Surgery [34]	2011	+				
19	▪ AHA/ACCF Secondary Prevention and Risk Reduction Therapy for Patients With Coronary and Other Atherosclerotic Vascular Disease: 2011 Update [13] ▪ AHA/ACC Guidelines for Secondary Prevention for Patients With Coronary and Other Atherosclerotic Vascular Disease: 2006 Update [14]	2011 2006	+				
20	▪ ASA/ACCF/AHA/AANN/AANS/ACR/ASNR/CNS/ SAIP/SCAI/SIR/SNIS/SVM/SVS Guideline on the Management of Patients With Extracranial Carotid and Vertebral Artery [20]	2011	+++	4	4	1	0
21	▪ ACCF/AHA/AATS/ACR/ASA/SCA/SCAI/SIR/STS/SVM Guidelines for the Diagnosis and Management of Patients With Thoracic Aortic Disease [21]	2010	+++	3	5	0	0
22	▪ ACCF/AHA Guideline for Assessment of Cardiovascular Risk in Asymptomatic Adults [18]	2010	+++	0	0	0	1
23	▪ ACC/AHA Guidelines for the Management of Adults With Congenital Heart Disease [19]	2008	+++	14	2	1	0
24	▪ ACC/AHA/ESC Guidelines for Management of Patients With Ventricular Arrhythmias and the Prevention of Sudden Cardiac Death [35]	2006	+++	0	1	0	0

**Table 2:** Class (Strength) of Recommendation [4]

Class of recommendation	Definition	Suggested phrases for writing recommendations
Class I (strong)	Benefit >> > Risk	• is recommended • is indicated / useful / effective / beneficial • should be performed / administered / other • Comparative-Effectiveness phrases: − Treatment / strategy A is recommended / indicated in preference to treatment B − Treatment A should be chosen over treatment B
Class IIa (Moderate)	Benefit > > Risk	• Is reasonable • Can be useful / effective / beneficial • Comparative-Effectiveness phrases − Treatment / strategy A is probably recommended / indicated in preference to treatment B − It is reasonable to choose treatment A over treatment B
Class IIb (Weak)	Benefit ≥ Risk	• May / might be reasonable • May / might be considered • Usefulness / effectiveness is unknown / unclear / uncertain or not well established
Class III: No benefit (Moderate)	Benefit = Risk	• Is not recommended • Is not indicated / useful / effective / beneficial • Should not be performed / administered / other
Class III: Harm (Strong)	Risk > Benefit	• Potentially harmful • Causes harm • Associated with excess morbidity / mortality • Should not be performed / administered / other

**Table 3:** Level of Evidence [4]

Level	Definition
Level A	• High quality evidence from more than 1 randomized controlled trial • Meta-analyses of high-quality randomized controlled trials • One or more randomized controlled trial corroborated by high-quality registry studies
Level B-R (randomized)	• Moderate-quality evidence from 1 or more randomized controlled trial • Meta-analyses of moderate-quality randomized controlled trials
Level B-NR (nonrandomized)	• Moderate-quality evidence from 1 or more well-designed well-executed nonrandomized studies, observational studies, or registry studies • Meta-analyses of such studies
Level C-LD (limited data)	• Randomized or nonrandomized observational or registry studies with limitations of design or execution • Meta-analyses of such studies • Physiological or mechanistic studies in human subjects
Level C-EO (expert opinion)	• Consensus of expert opinion based on clinical experience

## Results

In total 24 AHA/ACC guidelines ﻿were analyzed. For five guidelines, more recent updates were included in a combined analysis with the full guidelines (heart failure update 2017 and 2016 [5, 6], valve disease update 2017 [7], stable ischemic heart disease update 2014 [8], device-based therapy of cardiac rhythm abnormalities update 2012 [9]). There is one update from 2015 incorporating the previous STEMI and PCI guidelines [10–12]. In this case, all three documents were analyzed together, and the guidelines were counted as two separate cases. Two updates on secondary prevention were published during the inclusion period (2011 and 2006 [13, 14]), but no explicit original guideline. Both updates were analyzed and counted as one guideline. The “Guidelines on perioperative cardiovascular evaluation and care for non-cardiac surgery” were published twice (2014 and 2007 [15, 16]), but only the more recent version entered the quantitative analysis.

**Fig. 1:**
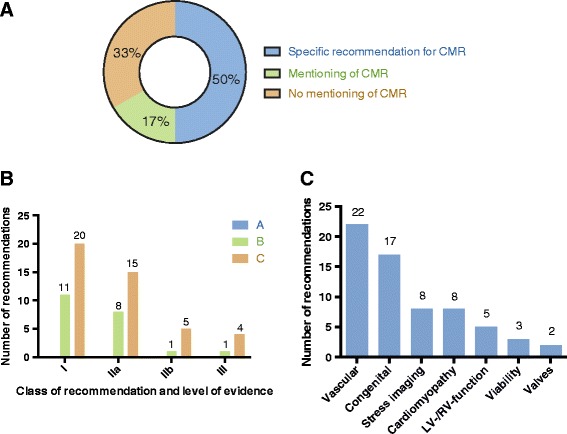
Panel **a** Categorization of the 24 analyzed AHA/ACC guidelines regarding the role of CMR. Panel **b** Distribution of the 65 specific recommendations for CMR in the 24 AHA/ACC guidelines regarding “class of recommendations” and “level of evidence”. Panel **c** Categorization of the 65 specific recommendations in the 24 AHA/ACC guidelines regarding the diagnostic target

Of the 24 analyzed AHA/ACC guidelines, 12 (50.0%) contain specific recommendations regarding the use of CMR (Table 1). Four guidelines (16.7%) principally mention scenarios in which CMR may be used, but without giving specific recommendations. Eight guidelines (33.3%) do not mention CMR at all. (Fig. 1).

The 12 guidelines with recommendations regarding the use of CMR contain in total 65 specific recommendations. These are 31 class-I recommendations, 23 class-IIa recommendations, 6 class-IIb recommendations and 5 class-III recommendations (Fig. 1). The 5 class-III recommendations stem from the guidelines concerning lower peripheral artery disease (*n* = 1) [4], stable ischemic heart disease (*n* = 3) [8, 17], and risk assessment in asymptomatic adults (n = 1) [18].

Most of the CMR recommendations have evidence level C (44/65; 67.7%), followed by level B (21/65; 32.3%). No CMR recommendations have evidence level A.

The four guidelines that contained the most recommendations for CMR, were the guidelines on adults with congenital heart disease (*n* = 17) [19], extracranial carotid and vertebral artery disease (*n* = 9) [20], thoracic aortic disease (*n* = 8) [21], and stable ischemic heart disease (n = 8) [8, 17].

Twenty-two of the 65 recommendations refer to vascular imaging, 17 recommendations refer to congenital heart disease, 8 to myocardial stress testing, 8 to cardiomyopathies, 5 to LV and RV function assessment, 3 to viability and 2 to valvular heart disease (Fig. 1).

Table 4 lists the 65 recommendations categorized by clinical scenario and diagnosis (following the style of the ESC guideline summary [3]).

- 2017 ACC/AHA/HFSA Focused Update of the 2013 ACCF/AHA Guideline for the Management of Heart Failure [5]
- 2016 ACC/AHA/HFSA Focused Update on New Pharmacological Therapy for Heart Failure: An Update of the 2013 ACCF/AHA Guideline for the Management of Heart Failure [6]
- 2013 ACCF/AHA Guideline for the Management of Heart Failure - A Report of the American College of Cardiology Foundation/American Heart Association Task Force on Practice Guidelines [22]

**Table 4:** Summary of clinical scenarios / diagnosis groups, where the AHA/ACC guidelines make recommendations regarding CMR

	Class	Level	Guideline
Suspected / stable coronary artery disease			
Noninvasive imaging to detect myocardial ischemia and viability is reasonable in heart failure and coronary artery disease	IIa	C	Heart failure [5, 6, 22]
Noninvasive imaging (stress nuclear/positron emission tomography, CMR, or stress echocardiography), cardiac CT angiography, or cardiac catheterization, including coronary arteriography, is useful to establish etiology of chronic secondary MR (stages B to D) and/or to assess myocardial viability, which in turn may influence management of functional MR.	I	C	Valve disease [7, 23]
Pharmacological stress with CMR can be useful for patients with an intermediate to high pretest probability of obstructive ischemic heart disease, who have an uninterpretable ECG and at least moderate physical functioning or no disabling comorbidity.	IIa	B	Stable CAD [8]
Pharmacological stress CMR is reasonable for patients with an intermediate to high pretest probability of ischemic heart disease, who are incapable of at least moderate physical functioning or have disabling comorbidity.	IIa	B	Stable CAD [8]
Echocardiography, radionuclide imaging, CMR, and cardiac CT are not recommended for routine assessment of LV function in patients with a normal ECG, no history of myocardial infarction, no symptoms or signs suggestive of heart failure, and no complex ventricular arrhythmias.	III	C	Stable CAD [8]
Routine reassessment (<1 year) of LV function with technologies such as echocardiography, radionuclide imaging, CMR, or cardiac CT is not recommended in patients with no change in clinical status and for whom no change in therapy is contemplated.	III	C	Stable CAD [8]
CMR with pharmacological stress is reasonable for risk assessment in patients with stable ischemic heart disease who are able to exercise to an adequate workload but have an uninterpretable ECG.	IIa	B	Stable CAD [8]
Pharmacological stress imaging (nuclear MPI, echocardiography, or CMR) or CCTA is not recommended for risk assessment in patients with stable ischemic heart disease who are able to exercise to an adequate workload and have an interpretable ECG.	III	C	Stable CAD [8]
Pharmacological stress CMR is reasonable for risk assessment in patients with stable ischemic heart disease who are unable to exercise to an adequate workload regardless of interpretability of ECG.	IIa	B	Stable CAD [8]
Acute coronary syndrome			
Imaging with ventriculography, echocardiography, or magnetic resonance imaging should be performed to confirm or exclude the diagnosis of stress (Takotsubo) cardiomyopathy.	I	B	NSTEMI [26]
Before coronary revascularization			
Viability assessment is reasonable before revascularization in heart failure patients with coronary artery disease	IIa	B	Heart failure [5, 6, 22]
Either exercise or pharmacological stress with imaging (nuclear MPI, echocardiography, or CMR) is recommended for risk assessment in patients with stable ischemic heart disease, who are being considered for revascularization of known coronary stenosis of unclear physiological significance.	I	B	Stable CAD [8]
Heart failure			
Radionuclide ventriculography or MRI can be useful to assess LVEF and volume	IIa	C	Heart failure [5, 6, 22]
MRI is reasonable when assessing myocardial infiltration or scar	IIa	B	Heart failure [5, 6, 22]
Ventricular arrhythmia			
MRI, cardiac computed tomography (CT), or radionuclide angiography can be useful in patients with ventricular arrhythmias when echocardiography does not provide accurate assessment of LV and RV function and/or evaluation of structural changes.	IIa	B	Ventricular arrhythmias [35]
Hypertrophic cardiomyopathy			
CMR imaging is indicated in patients with suspected HCM when echocardiography is inconclusive for diagnosis.	I	B	HCM [33]
CMR imaging is indicated in patients with known HCM when additional information that may have an impact on management or decision making regarding invasive management, such as magnitude and distribution of hypertrophy or anatomy of the mitral valve apparatus or papillary muscles, is not adequately defined with echocardiography.	I	B	HCM [33]
CMR imaging is reasonable in patients with HCM to define apical hypertrophy and/or aneurysm if echocardiography is inconclusive.	IIa	B	HCM [33]
In selected patients with known HCM, when SCD risk stratification is inconclusive after documentation of the conventional risk factors, CMR imaging with assessment of late gadolinium enhancement (LGE) may be considered in resolving clinical decision making.	IIb	C	HCM [33]
The usefulness of the following potential SCD risk modifiers is unclear but might be considered in selected patients with HCM for whom risk remains borderline after documentation of conventional risk factors: CMR imaging with LGE.	IIb	C	HCM [33]
Athlete’s heart			
Extended monitoring (including MRI) can be beneficial for athletes with unexplained exertional syncope after an initial cardiovascular evaluation.	IIa	C-LD	Syncope [24]
Storage disease			
CMR imaging may be considered in patients with LV hypertrophy and the suspicion of alternative diagnoses to HCM, including cardiac amyloidosis, Fabry disease, and genetic phenocopies such as LAMP2 cardiomyopathy.	IIb	C	HCM [33]
Vascular disease			
Aortic magnetic resonance angiography or CT angiography is indicated in patients with a bicuspid aortic valve when morphology of the aortic sinuses, sinotubular junction, or ascending aorta cannot be assessed accurately or fully by echocardiography. (Level of Evidence: C)	I	C	Valve disease [7, 23]
Serial evaluation of the size and morphology of the aortic sinuses and ascending aorta by echocardiography, CMR, or CT angiography is recommended in patients with a bicuspid aortic valve and an aortic diameter greater than 4.0 cm, with the examination interval determined by the degree and rate of progression of aortic dilation and by family history. In patients with an aortic diameter greater than 4.5 cm, this evaluation should be performed annually.	I	C	Valve disease [7, 23]
Duplex ultrasound, computed tomography angiography (CTA), or magnetic resonance angiography (MRA) of the lower extremities is useful to diagnose anatomic location and severity of stenosis for patients with symptomatic peripheral artery disease in whom revascularization is considered	I	B-NR	Peripheral Artery Disease [4]
Invasive and noninvasive angiography (ie, CTA, MRA) should not be performed for the anatomic assessment of patients with asymptomatic peripheral artery disease.	III	B-R	Peripheral Artery Disease [4]
In patients with acute, focal ischemic neurological symptoms corresponding to the territory supplied by the left or right internal carotid artery, magnetic resonance angiography (MRA) or computed tomography angiography (CTA) is indicated to detect carotid stenosis when sonography either cannot be obtained or yields equivocal or otherwise nondiagnostic results.	I	C	Carotid and vertebral artery [20]
When an extracranial source of ischemia is not identified in patients with transient retinal or hemispheric neurological symptoms of suspected ischemic origin, CTA, MRA, or selective cerebral angiography can be useful to search for intracranial vascular disease.	IIa	C	Carotid and vertebral artery [20]
When the results of initial noninvasive imaging are inconclusive, additional examination by use of another imaging method is reasonable. In candidates for revascularization, MRA or CTA can be useful when results of carotid duplex ultrasonography are equivocal or indeterminate.	IIa	C	Carotid and vertebral artery [20]
When intervention for significant carotid stenosis detected by carotid duplex ultrasonography is planned, MRA, CTA, or catheter-based contrast angiography can be useful to evaluate the severity of stenosis and to identify intrathoracic or intracranial vascular lesions that are not adequately assessed by duplex ultrasonography.	IIa	C	Carotid and vertebral artery [20]
MRA without contrast is reasonable to assess the extent of disease in patients with symptomatic carotid atherosclerosis and renal insufficiency or extensive vascular calcification.	IIa	C	Carotid and vertebral artery [20]
When complete carotid arterial occlusion is suggested by duplex ultrasonography, MRA, or CTA in patients with retinal or hemispheric neurological symptoms of suspected ischemic origin, catheter-based contrast angiography may be considered to determine whether the arterial lumen is sufficiently patent to permit carotid revascularization.	IIb	C	Carotid and vertebral artery [20]
Noninvasive imaging by CTA or MRA for detection of vertebral artery disease should be part of the initial evaluation of patients with neurological symptoms referable to the posterior circulation and those with subclavian steal syndrome.	I	C	Carotid and vertebral artery [20]
In patients whose symptoms suggest posterior cerebral or cerebellar ischemia, MRA or CTA is recommended rather than ultrasound imaging for evaluation of the vertebral arteries.	I	C	Carotid and vertebral artery [20]
Contrast-enhanced CTA, MRA, and catheter-based contrast angiography are useful for diagnosis of cervical artery dissection.	I	C	Carotid and vertebral artery [20]
Urgent and definitive imaging of the aorta using transesophageal echocardiogram, computed tomographic imaging, or magnetic resonance imaging is recommended to identify or exclude thoracic aortic dissection in patients at high risk for the disease by initial screening.	I	B	Thoracic aorta [21]
The initial evaluation of Takayasu arteritis or giant cell arteritis should include thoracic aorta and branch vessel computed tomographic imaging or magnetic resonance imaging to inves- tigate the possibility of aneurysm or occlusive disease in these vessels.	I	C	Thoracic aorta [21]
For patients with isolated aortic arch aneurysms less than 4.0 cm in diameter, it is reasonable to reimage using computed tomographic imaging or magnetic resonance imaging, at 12- month intervals, to detect enlargement of the aneurysm.	IIa	C	Thoracic aorta [21]
For patients with isolated aortic arch aneurysms 4.0 cm or greater in diameter, it is reasonable to reimage using computed tomographic imaging or magnetic resonance imaging, at 6-month intervals, to detect enlargement of the aneurysm.	IIa	C	Thoracic aorta [21]
For imaging of pregnant women with aortic arch, descending, or abdominal aortic dilatation, magnetic resonance imaging (without gadolinium) is recommended over computed tomographic imaging to avoid exposing both the mother and fetus to ionizing radiation. Transesophageal echocardiogram is an option for imaging of the thoracic aorta.	I	C	Thoracic aorta [21]
Computed tomographic imaging or magnetic resonance imaging of the thoracic aorta is reasonable after a Type A or B aortic dissection or after prophylactic repair of the aortic root/ ascending aorta.	IIa	C	Thoracic aorta [21]
Computed tomographic imaging or magnetic resonance imaging of the aorta is reasonable at 1, 3, 6, and 12 months postdissection and, if stable, annually thereafter so that any threatening enlargement can be detected in a timely fashion.	IIa	C	Thoracic aorta [21]
If a thoracic aortic aneurysm is only moderate in size and remains relatively stable over time, magnetic resonance imaging instead of computed tomographic imaging is reasonable to minimize the patient’s radiation exposure.	IIa	C	Thoracic aorta [21]
MRI for detection of vascular plaque is not recommended for cardiovascular risk assessment in asymptomatic adults.	III	C	Risk assessment [18]
Valvular heart disease			
CMR is indicated in patients with moderate or severe AR (stages B, C, and D) and suboptimal echocardiographic images for the assessment of LV systolic function, systolic and diastolic volumes, and measurement of AR severity.	I	B	Valve disease [7, 23]
CMR is indicated in patients with chronic primary MR to assess LV and RV volumes, function, or MR severity and when these issues are not satisfactorily addressed by TTE.	I	B	Valve disease [7, 23]
CMR or real-time 3D echocardiography may be considered for assessment of right ventricular systolic function and systolic and diastolic volumes in patients with severe tricuspid regurgitation (stages C and D) and suboptimal 2D echocardiograms.	IIb	C	Valve disease [7, 23]
Congenital heart disease			
Diagnostic and interventional procedures, including imaging (ie, echocardiography, MRI, or CT, advanced cardiac catheterization, and electrophysiology procedures for adults with complex and moderate CHD should be performed in a regional ACHD center with appropriate experience in CHD and in a laboratory with appropriate personnel and equipment. Personnel performing such procedures should work as part of a team with expertise in the surgical and transcatheter management of patients with CHD.	I	C	Congenital heart disease [19]
(In bicuspid aortic valve disease) MRI/CT can be beneficial to add important information about the anatomy of the thoracic aorta.	IIa	C	Congenital heart disease [19]
(In bicuspid aortic valve disease) MRI may be beneficial in quantifying aortic regurgitation when other data are ambiguous or borderline.	IIb	C	Congenital heart disease [19]
(In supravalvular aortic stenosis) TTE and/or TEE with Doppler and either MRI or CT should be performed to assess the anatomy of the LVOT, the ascending aorta, coronary artery anatomy and flow, and main and branch pulmonary artery anatomy and flow.	I	C	Congenital heart disease [19]
Every patient with coarctation (repaired or not) should have at least 1 cardiovascular MRI or CT scan for complete evaluation of the thoracic aorta and intracranial vessels.	I	B	Congenital heart disease [19]
Evaluation of the coarctation repair site by MRI/CT should be performed at intervals of 5 years or less, depending on the specific anatomic findings before and after repair.	I	C	Congenital heart disease [19]
Patients with suspected supravalvular, branch, or peripheral pulmonary stenosis should have baseline imaging with echocardiography-Doppler plus 1 of the following: MRI angiography, CT angiography, or contrast angiography.	I	C	Congenital heart disease [19]
(In congenital coronary anomalies of ectopic arterial origin) CT or MRA is useful as the initial screening method in centers with expertise in such imaging.	I	B	Congenital heart disease [19]
(In suspicion of a coronary arteriovenous fistula), if a continuous murmur is present, its origin should be defined either by echocardiography, MRI, CT angiography, or cardiac catheterization.	I	C	Congenital heart disease [19]
The evaluation of all ACHD patients with suspected pulmonary arterial hypertension should include noninvasive assessment of cardiovascular anatomy and potential shunting, as detailed below: Diagnostic cardiovascular imaging via TTE, TEE, MRI, or CT as appropriate.	I	C	Congenital heart disease [19]
Patients with tetralogy of Fallot should have echocardiographic examinations and/or MRIs performed by staff with expertise in ACHD.	I	C	Congenital heart disease [19]
Additional imaging with TEE, CT, or MRI, as appropriate, should be performed in a regional ACHD center to evaluate the great arteries and veins, as well as ventricular function, in patients with prior atrial baffle repair of d-TGA.	I	B	Congenital heart disease [19]
Periodic MRI or CT can be considered appropriate to evaluate the anatomy and hemodynamics in more detail in patients with prior arterial switch operation.	IIa	C	Congenital heart disease [19]
(In congenitally corrected transposition of the great arteries), echocardiography-Doppler study and/or MRI should be performed yearly or at least every other year by staff trained in imaging complex CHD.	I	C	Congenital heart disease [19]
The following diagnostic evaluations are recommended for patients with congenitally corrected transposition of the great arteries: ECG, chest x-ray, echocardiography-Doppler study, MRI, exercise testing.	I	C	Congenital heart disease [19]
(In patients with prior repair of congenitally corrected transposition of the great arteries), echocardiography-Doppler study and/or MRI should be performed yearly or at least every other year by staff trained in imaging complex CHD.	I	C	Congenital heart disease [19]
All patients with prior Fontan type of repair should have periodic echocardiographic and/or magnetic resonance examinations performed by staff with expertise in ACHD.	I	C	Congenital heart disease [19]
Syncope			
Computed tomography (CT) or magnetic resonance imaging (MRI) may be useful in selected patients presenting with syncope of suspected cardiac etiology.	IIb	B-NR	Syncope [24]

Class = class of recommendation

Level = level of evidence

NSTEMI = non-ST-elevation myocardial infarction

CAD = coronary artery disease

HCM = hypertrophic cardiomyopathy

The most recent full AHA/ACC guideline regarding heart failure was published in 2013 and updated in 2016 and 2017. In the 2013 full version, under the topic “Initial and serial evaluation of the heart failure patient”, specific recommendations for the use of CMR are defined, in particular regarding assessment of LV function, perfusion and viability (Table 5). CMR is additionally mentioned as an alternative to echocardiography, as CMR “assesses LV volume and EF measurements at least as accurately as echocardiography” (page 19). Furthermore, its potential to provide “additional information about myocardial perfusion, viability, and fibrosis can help identify heart failure etiology and assess prognosis” (page 19) is highlighted. Finally, the use of CMR in known or suspected congenital heart diseases is indicated as “CMR provides high anatomical resolution of all aspects of the heart and surrounding structure” (page 19). Under the heading “Cardiac structural abnormalities and other causes of heart failure”, CMR is recommended in subjects with known or suspected cardiac sarcoidosis, as CMR “can identify cardiac involvement with patchy areas of myocardial inflammation and fibrosis” (page 14).

- 2017 AHA/ACC Focused Update of the 2014 AHA/ACC Guideline for the Management of Patients With Valvular Heart Disease [7]
- 2014 AHA/ACC Guideline for the Management of Patients With Valvular Heart Disease [23]

**Table 5:** Recommendations for CMR in heart failure

Recommendations for non-invasive cardiac imaging in heart failure	Class^a^	Level^b^	Page
Radionuclide ventriculography or MRI can be useful to assess LVEF and volume	IIa	C	18
Noninvasive imaging to detect myocardial ischemia and viability is reasonable in heart failure and coronary artery disease	IIa	C	18
Viability assessment is reasonable before revascularization in heart failure patients with coronary artery disease	IIa	B	18
MRI is reasonable when assessing myocardial infiltration or scar	IIa	B	18

^a^Class of recommendation

^b^Level of evidence

In patients with suspected valvular heart disease, echocardiography is the cornerstone of the diagnostic algorithm. The guideline adds that generally “Other ancillary testing such as transesophageal echocardiography (TEE), computed tomography (CT) or cardiac magnetic resonance (CMR) imaging, stress testing, and diagnostic hemodynamic cardiac catheterization may be required to determine the optimal treatment for a patient with valvular heart disease” (page 7).

Specifically, in *aortic stenosis*, “CMR imaging shows promise for evaluation of severity of aortic stenosis, but is not widely available” (page 17). In *aortic regurgitation*, “CMR imaging provides accurate measures of regurgitant volume and regurgitant fraction... as well as assessment of aortic morphology, LV volume, and LV systolic function. In addition to its value in patients with suboptimal echocardiographic data, CMR is useful for evaluating patients in whom there is discordance between clinical assessment and severity of aortic regurgitation by echocardiography. CMR measurement of regurgitant severity is less variable than echocardiographic measurement” (page 29). This approach is expressed as a Class I, Level B-recommendation (Table 6). In subjects with aortic regurgitation and chronic aortic disease, “CMR imaging is useful ..., but is rarely used in unstable patients with suspected dissection” (page 27). For the subset of patients with bicuspid aortic valve disease, the guideline defines several specific recommendations about the use of CMR to assess the thoracic aorta (Table 6). In this context, the guideline states that “Magnetic resonance angiography or chest CT angiography provide accurate diameter measurements when aligned perpendicular to the long axis of the aorta. Advantages of magnetic resonance angiography and CT angiography compared with TTE include higher spatial (but lower temporal) resolution and the ability to display a 3D reconstruction of the entire length of the aorta”(page 33). Furthermore, the guideline emphasizes that “Magnetic resonance angiography imaging is preferred over CT angiography imaging, when possible, because of the absence of ionizing radiation exposure in patients who likely will have multiple imaging studies over their lifetime” (page 33).

**Table 6:** Recommendations for CMR in valvular heart disease

	Class^a^	Level^b^	Page
Aortic regurgitation			
CMR is indicated in patients with moderate or severe AR (stages B, C, and D) and suboptimal echocardiographic images for the assessment of LV systolic function, systolic and diastolic volumes, and measurement of AR severity.	I	B	29
Bicuspid aortic valve disease			
Aortic magnetic resonance angiography or CT angiography is indicated in patients with a bicuspid aortic valve when morphology of the aortic sinuses, sinotubular junction, or ascending aorta cannot be assessed accurately or fully by echocardiography.	I	C	32
Serial evaluation of the size and morphology of the aortic si- nuses and ascending aorta by echocardiography, CMR, or CT angiography is recommended in patients with a bicuspid aortic valve and an aortic diameter greater than 4.0 cm, with the examination interval determined by the degree and rate of progression of aortic dilation and by family history. In patients with an aortic diameter greater than 4.5 cm, this evaluation should be performed annually.	I	C	33
Mitral regurgitation			
CMR is indicated in patients with chronic primary MR to assess LV and RV volumes, function, or MR severity and when these issues are not satisfactorily addressed by TTE.	I	B	43
Noninvasive imaging (stress nuclear/positron emission tomog- raphy, CMR, or stress echocardiography), cardiac CT angiography, or cardiac catheterization, including coronary arteriography, is useful to establish etiology of chronic secondary MR (stages B to D) and/or to assess myocardial viability, which in turn may influence management of functional MR.	I	C	50
Tricuspid regurgitation			
CMR or real-time 3D echocardiography may be considered for assessment of right ventricular systolic function and systolic and diastolic volumes in patients with severe tricuspid regurgitation (stages C and D) and suboptimal 2D echocardiograms.	IIb	C	54

^a^Class of recommendation

^b^Level of evidence

In *mitral regurgitation (MR)*, “in cases where TTE image quality is poor, CMR may be of value in MR evaluation. CMR produces highly accurate data on LV volumes, RV volumes, and LVEF, and an estimation of MR severity, but outcome data using CMR volumes is pending. CMR is less helpful in establishing mitral pathoanatomy” (pages 43–44; Table 6). Furthermore, “Three-dimensional echocardiography, strain imaging, or CMR may add more accurate assessment of the LV response in the future” (page 43).

In *tricuspid regurgitation*, “Both CMR and real-time 3D echocardiography may provide more accurate assessment of right ventricular volumes and systolic function, as well as annular dimension and the degree of leaflet tethering. CMR may be the ideal modality in young asymptomatic patients with severe tricuspid regurgitation to assess initial and serial measurements of right ventricular size and systolic function” (Table 6).

- 2017 ACC/AHA/HRS Guideline for the Evaluation and Management of Patients With Syncope [24]

“Imaging modalities, including CT and MRI, are usually reserved for selected patients presenting with syncope, especially when other noninvasive means are inadequate or inconclusive. These modalities offer superior spatial resolution in delineating cardiovascular anatomy (e.g., in patients with structural, infiltrative, or congenital heart disease. ... MRI is useful when there is a suspicion of ARVC or cardiac sarcoidosis” (page 25). In athletes, “imaging may include echocardiography or MRI as required” (page 64). The specific recommendations for CMR in patients with syncope are shown in Table 7.

- 2016 AHA/ACC Guideline on the management of patients with lower extremity peripheral artery disease [4]

**Table 7:** Recommendations for cardiac imaging in syncope

Recommendations for cardiac imaging in syncope	Class^a^	Level^b^	Page
Computed tomography (CT) or magnetic resonance imaging (MRI) may be useful in selected patients presenting with syncope of suspected cardiac etiology.	IIb	B-NR	25
Extended monitoring (including MRI) can be beneficial for athletes with unexplained exertional syncope after an initial cardiovascular evaluation.	IIa	C-LD	64

^a^Class of recommendation

^b^Level of evidence

This guideline contains two recommendations for CMR under the heading “3.3. Imaging for anatomic assessment” (Table 8). Furthermore, Fig. 1 in the guideline (“Diagnostic testing for suspected peripheral artery disease”) and figure 2 in the guideline (“Diagnostic testing for suspected critical limb ischemia”) include CMR as part of the diagnostic algorithm.

**Table 8:** Recommendations for CMR in peripheral artery disease

Recommendations for imaging for anatomic assessment	Class^a^	Level^b^	Page
Duplex ultrasound, computed tomography angiography (CTA), or magnetic resonance angiography (MRA) of the lower extremities is useful to diagnose anatomic location and severity of stenosis for patients with symptomatic peripheral artery disease in whom revascularization is considered	I	B-NR	24
Invasive and noninvasive angiography (ie, CTA, MRA) should not be performed for the anatomic assessment of patients with asymptomatic peripheral artery disease.	III	B-R	25

^a^Class of recommendation

^b^Level of evidence

For symptomatic patients with peripheral artery disease, in whom revascularization is considered, additional imaging with duplex ultrasonography, CTA, or MRA is useful to develop an individualized treatment plan. The guidelines state, “all 3 of these noninvasive imaging methods have good sensitivity and specificity as compared with invasive angiography” (page 24). CMR is characterized by superior spatial resolution compared to ultrasound. The guideline also discusses the issue that gadolinium contrast, used frequently in CMR angiography studies, can confer risk of nephrogenic systemic sclerosis in patients with advanced renal dysfunction. Generally, the choice of the examination should be determined in an individualized approach to the anatomic assessment for each patient, including risk-benefit assessment of each study type (page 24).

Furthermore, the guideline emphasized that angiography, either noninvasive or invasive, should not be performed for the anatomic assessment of patients with peripheral artery disease without leg symptoms because delineation of anatomy will not change treatment for this population (page 25), expressed as a III-B recommendation.

- 2015 ACC/AHA/HRS Guideline for the management of adult patients with supraventricular tachycardia [25]

This guideline does not mention CMR.

- 2015 ACC/AHA/SCAI Focused Update on Primary Percutaneous Coronary Intervention for Patients With ST-Elevation Myocardial Infarction: An Update of the 2011 ACCF/AHA/SCAI Guideline for Percutaneous Coronary Intervention and the 2013 ACCF/AHA Guideline for the Management of ST-Elevation Myocardial Infarction [10]
- 2013 ACCF/AHA Guideline for the Management of ST-Elevation Myocardial Infarction [11]
- 2011 ACCF/AHA/SCAI Guideline for Percutaneous Coronary Intervention [12]

The 2015 update and the 2013 STEMI guideline do not mention CMR. In the 2011 percutaneous coronary intervention guideline, CMR is mentioned in the text as a diagnostic tool to detect periprocedural myocardial infarction (page 40).

- 2014 ACC/AHA Guideline on Perioperative Cardiovascular Evaluation and Management of Patients Undergoing Noncardiac Surgery [15]
- 2007 ACC/AHA Guidelines on Perioperative Cardiovascular Evaluation and Care for Noncardiac Surgery [16]

In subjects undergoing non-cardiac surgery, the appropriate preoperative use of non-invasive stress testing is discussed. There are several specific recommendations in the guideline covering different clinical scenarios. The only recommended stress (exercise or pharmacology) tests with imaging are nuclear myocardial perfusion and dobutamine stress echocardiography (pages 18–20). Regarding CMR it is stated, “There are insufficient data to support the use of dobutamine stress magnetic resonance imaging in preoperative risk assessment” (page 20). Under the heading “future research directions”, the document states, “Diagnostic cardiovascular testing continues to evolve, with newer imaging modalities being developed, such as ... cardiac magnetic resonance imaging. The value of these modalities in preoperative screening is uncertain and warrants further study” (page 35).

- 2014 ACC/AHA/AATS/PCNA/SCAI/STS Focused Update of the Guideline for the Diagnosis and Management of Patients With Stable Ischemic Heart Disease [8]
- 2012 ACCF/AHA/ACP/AATS/PCNA/SCAI/STS Guideline for the Diagnosis and Management of Patients With Stable Ischemic Heart Disease [17]

In the 2014 update, CMR is not mentioned. One of the central aspects of the 2012 full guideline is the appropriate use of noninvasive stress testing to assess coronary artery disease. Nuclear perfusion imaging and stress-echocardiography are generally regarded as first choices; however, CMR is included both in the text and in the diagnostic algorithms and in the specific recommendations, underlying its increasing importance.

The guideline is separated into several parts, with the first part focusing on the initial diagnosis of stable ischemic heart disease. Stress CMR is included both in figure 2 of the guideline (page 12) illustrating the diagnostic algorithm, and in table 11 of the guideline (page 23) that summarizes the recommendation level for all available diagnostic tests. Two specific recommendations for stress CMR are expressed (Table 9). A subchapter (page 25) summarizes the current evidence regarding the diagnostic accuracy of pharmacological stress CMR wall motion / perfusion imaging, and underlines the complementary information provided by late gadolinium enhancement scar imaging. In addition, under the subheading “cost effectiveness”, data from the EuroCMR registry are mentioned, which provided evidence that “CMR can improve patient management” by reducing the number of indicated coronary angiographies (page 22). Finally, the idea of imaging the coronary anatomy by CMR angiography is discussed in a subchapter (page 26), pointing at the principal feasibility in studies, but also underlining its variable diagnostic accuracy and limited widespread use. On page 21, potential limitations of CMR (claustrophobia, implanted devices, nephrogenic systemic fibrosis) are mentioned.

**Table 9:** Recommendations for CMR for the diagnosis of stable coronary artery disease

Diagnosis stable coronary artery disease	Class^a^	Level^b^	Page
Pharmacological stress with CMR can be useful for patients with an intermediate to high pretest probability of obstructive ischemic heart disease, who have an uninterpretable ECG and at least moderate physical functioning or no disabling comorbidity.	IIa	B	22
Pharmacological stress CMR is reasonable for patients with an intermediate to high pretest probability of ischemic heart disease, who are incapable of at least moderate physical functioning or have disabling comorbidity.	IIa	B	24

^a^Class of recommendation

^b^Level of evidence

A second part of the guideline deals with risk stratification in known coronary artery disease. The comprehensive information gained by CMR is pronounced: CMR “accurately measures LV performance and provides insight into myocardial and valvular structures. Use of delayed hyperenhancement techniques can identify otherwise undetected scarred as well as viable myocardium.” (page 30). The use of CMR to risk stratify subjects with coronary artery disease is integrated both in figure 3 of the guideline (page 13) containing the corresponding diagnostic algorithm, in table 12 of the guideline (page 31) that summarizes the recommendation level for all diagnostic tests and in tables 20 and 21 of the guideline (page 77–78) that summarize the tests for follow-up. Several scenarios where CMR is or is not recommended are reflected in specific recommendations (Table 10). A subchapter summarizes the evidence for pharmacological stress CMR to gain prognostic information (page 33). It concludes: 1. “A normal stress CMR study with either vasodilator myocardial perfusion or inotropic stress cine imaging is associated with a low annual rate of cardiac death or myocardial infarction. 2. Detection of myocardial ischemia... and LGE imaging of infarction appear to provide complementary information. 3. An abnormal stress CMR with evidence of ischemia is associated with elevated likelihood of cardiac death or myocardial infarct”. Another subchapter about “Future developments” (page 81) predicts increasing use of CMR in stable coronary artery disease in the future, being supported by upcoming technological developments, like image acquisition acceleration.

- 2014 AHA/ACC Guideline for the Management of Patients With Non–ST-Elevation Acute Coronary Syndromes [26]

**Table 10:** Recommendations for CMR for risk stratification and follow-up in stable coronary artery disease

	Class^a^	Level^b^	Page
Resting imaging to assess cardiac structure and function			
Echocardiography, radionuclide imaging, CMR, and cardiac CT are not recommended for routine assessment of LV function in patients with a normal ECG, no history of myocardial infarction, no symptoms or signs suggestive of heart failure, and no complex ventricular arrhythmias.	III	C	29
Routine reassessment (<1 year) of LV function with technologies such as echocardiography, radionuclide imaging, CMR, or cardiac CT is not recommended in patients with no change in clinical status and for whom no change in therapy is contemplated.	III	C	31
Risk assessment in patients able to exercise			
CMR with pharmacological stress is reasonable for risk assessment in patients with stable ischemic heart disease who are able to exercise to an adequate workload but have an uninterpretable ECG.	IIa	B	30
Pharmacological stress imaging (nuclear MPI, echocardiography, or CMR) or CCTA is not recommended for risk assessment in patients with stable ischemic heart disease who are able to exercise to an adequate workload and have an interpretable ECG.	III	C	30
Risk assessment in patients unable to exercise			
Pharmacological stress CMR is reasonable for risk assessment in patients with stable ischemic heart disease who are unable to exercise to an adequate workload regardless of interpretability of ECG.	IIa	B	30
Risk assessment regardless of patients’ ability to exercise			
Either exercise or pharmacological stress with imaging (nuclear MPI, echocardiography, or CMR) is recommended for risk assessment in patients with stable ischemic heart disease, who are being considered for revascularization of known coronary stenosis of unclear physiological significance.	I	B	31

^a^Class of recommendation

^b^Level of evidence

This guideline deals with CMR in two scenarios. First, to determine early invasive versus ischemia-guided strategy in patients with NSTEMI, the guideline mentions that - among other factors – “noninvasive stress test findings, including magnetic resonance imaging, may aid in the identification of high-risk patients who could benefit from an invasive strategy” (page 28). Second, in patients presenting as NSTEMI but having angiographically normal coronary arteries, the guideline mentions that “Myocarditis may present with electrocardiographic and biomarker findings similar to ACS and can be distinguished by magnetic resonance imaging” (page 49). Furthermore, the recommendation to use CMR to assess the presence of stress (Takotsubo) cardiomyopathy is given (Table 11).

- 2014 AHA/ACC/HRS Guideline for the Management of Patients With Atrial Fibrillation [27]

**Table 11:** Recommendations for CMR in NSTEMI with angiographically normal coronary arteries

*Stress (Takotsubo) cardiomyopathy*	Class^a^	Level^b^	Page
Imaging with ventriculography, echocardiography, or magnetic resonance imaging should be performed to confirm or exclude the diagnosis of stress (Takotsubo) cardiomyopathy.	I	B	49

^a^Class of recommendation

^b^Level of evidence

CMR is mentioned in the text in the chapter “Mechanisms of atrial fibrillation and pathophysiology / Atrial structural abnormalities”: “Late gadolinium-enhancement magnetic resonance imaging is used to image and quantitate atrial fibrosis noninvasively. Human studies show a strong correlation between regions of low voltage on electroanatomic mapping and areas of late enhancement on magnetic resonance imaging. Preliminary results suggest that the severity of atrial fibrosis correlates with the risk of stroke and decreased response to catheter ablation” (page 9–10).

- 2013 AHA/ACC/TOS Guideline for the Management of Overweight and Obesity in Adults [28]

CMR is not mentioned in this guideline.

- 2013 AHA/ACC Guideline on Lifestyle Management to Reduce Cardiovascular Risk [29]

CMR is not mentioned in this guideline.

- 2013 ACC/AHA Guideline on the Treatment of Blood Cholesterol to Reduce Atherosclerotic Cardiovascular Risk in Adults [30]

CMR is not mentioned in this guideline.

- 2013 ACC/AHA Guideline on the Assessment of Cardiovascular Risk [31]

CMR is not mentioned in this guideline.

- 2012 ACCF/AHA/HRS Focused Update Incorporated Into the ACCF/AHA/HRS 2008 Guidelines for Device-Based Therapy of Cardiac Rhythm Abnormalities [9]
- 2008 ACC/AHA/HRS Guidelines for Device-Based Therapy of Cardiac Rhythm Abnormalities [32]

In both issues, CMR is mentioned once in the text as a diagnostic tool to detect non-compaction of the left ventricle (page 36).

- 2011 ACCF/AHA Guideline for the Diagnosis and Treatment of Hypertrophic Cardiomyopathy [33]

This guideline includes CMR in several text passages as well as in specific recommendations for both diagnosis and risk stratification.

“The clinical diagnosis of HCM is conventionally made with cardiac imaging, at present most commonly with 2-dimensional echocardiography and increasingly with CMR” (page 10). “In terms of LV wall-thickness measurements, CMR is now used with increasing frequency, and the writing group presumes that data with this latter modality will increasingly emerge” (page 6). “Compared with other noninvasive cardiac imaging modalities, CMR provides superior spatial resolution with sharp contrast between blood and myocardium, as well as complete tomographic imaging of the entire LV myocardium and therefore the opportunity to more accurately characterize the presence, distribution, and extent of LV hypertrophy in HCM” (page 14). “There remain patients in whom the diagnosis of HCM is suspected but the echocardiogram is inconclusive, mostly because of suboptimal imaging from poor acoustic windows or when hypertrophy is localized to regions of the LV myocardium not well visualized by echocardiography... (predominantly anterolateral wall... or confined to the apex)”. (page 15). “Similarly, in the subgroup of patients with HCM who develop apical aneurysms, CMR can more readily detect the presence of an aneurysm” (page 15). Therefore, CMR is also mentioned as a diagnostic tool for HCM screening (page 11). Finally, other diseases may have overlapping phenotypes. CMR with LGE contributes to differentiate various forms of LV hypertrophy, like HCM, Anderson-Fabry disease or cardiac amyloidosis (page 15). Table 12 summarizes the specific recommendations for the use of CMR in HCM.

**Table 12:** Recommendations for CMR in HCM

	Class^a^	Level^b^	Page
CMR for the diagnosis of HCM			
CMR imaging is indicated in patients with suspected HCM when echocardiography is inconclusive for diagnosis.	I	B	14
CMR imaging is indicated in patients with known HCM when additional information that may have an impact on management or decision making regarding invasive management, such as magnitude and distribution of hypertrophy or anatomy of the mitral valve apparatus or papillary muscles, is not adequately defined with echocardiography.	I	B	14
CMR imaging is reasonable in patients with HCM to define apical hypertrophy and/or aneurysm if echocardiography is inconclusive.	IIa	B	14
CMR imaging may be considered in patients with LV hypertrophy and the suspicion of alternative diagnoses to HCM, including cardiac amyloidosis, Fabry disease, and genetic phenocopies such as LAMP2 cardiomyopathy.	IIb	C	14
CMR for risk stratification in HCM			
In selected patients with known HCM, when SCD risk stratification is inconclusive after documentation of the conventional risk factors, CMR imaging with assessment of late gadolinium enhancement (LGE) may be considered in resolving clinical decision making.	IIb	C	14
The usefulness of the following potential SCD risk modifiers is unclear but might be considered in selected patients with HCM for whom risk remains borderline after documentation of conventional risk factors: CMR imaging with LGE.	IIb	C	27

^a^Class of recommendation

^b^Level of evidence

In addition to its diagnostic value, the use of CMR for risk stratification in HCM is also discussed in the guideline. “Patients with HCM with evidence of LGE on CMR imaging tend to have more markers of risk of sudden cardiac death, such as non-sustained VT, than patients without LGE” (page 15). It is a plausible and attractive concept that areas of LGE... could represent a substrate for the generation of malignant ventricular tachyarrhythmias in HCM and thus a marker for risk of SCD. Several studies have addressed this issue and have reported either trends in such a direction or significant associations between the presence of LGE ... and cardiac outcome events. “However, there is insufficient evidence at this time to support a significant association between the extent of LGE and outcome. ... Nonetheless, the present cross-data would support a potential role of ... LGE as an arbitrator to consider in clinical decision making for primary prevention ICDs in patients in whom high-risk status for sudden cardiac death remains uncertain after assessment of conventional risk factors” (page 15). This assessment is reflected in a specific recommendation (Table 12).

Finally, as HCM is regarded as a complex disease entity, the writing committee emphasizes establishing clinical excellence centers, including access to CMR imaging (page 8).

- 2011 ACCF/AHA Guideline for Coronary Artery Bypass Graft Surgery [34]

CMR is not mentioned in this guideline.

- 2011 AHA/ACCF Secondary Prevention and Risk Reduction Therapy for Patients With Coronary and Other Atherosclerotic Vascular Disease: 2011 Update [13]
- 2006 AHA/ACC Guidelines for Secondary Prevention for Patients With Coronary and Other Atherosclerotic Vascular Disease: 2006 Update [14]

CMR is not mentioned in these guideline updates.

- 2011 ASA/ACCF/AHA/AANN/AANS/ACR/ASNR/CNS/ SAIP/SCAI/SIR/SNIS/SVM/SVS Guideline on the Management of Patients With Extracranial Carotid and Vertebral Artery [20]

The guideline contains several specific recommendations for the use of MR angiography to assess both carotid and vertebral arteries (Table 13). In addition, the strength and limitations of MRA are described in detail. Among other aspects, the NASCET stenosis grade based on angiographic criteria corresponds well to sonography, CT angiography and MR angiography, although the latter may overestimate the severity of stenosis (page 17). It is also stated that patients with a high pretest probability of disease may be examined initially by MR angiography or CT angiography to more completely evaluate the cerebral vessels distal to the aortic arch, because sonographic imaging alone does not provide assessment of intrathoracic or intracranial lesions beyond the limited range of the ultrasound probe (page 21). After carotid stenting, imaging by CT angiography or MR angiography may also be helpful for surveillance, particularly “when Doppler interrogation is difficult because of a superior anatomic location of the region of interest” (page 41).

- 2010 ACCF/AHA/AATS/ACR/ASA/SCA/SCAI/SIR/STS/SVM Guidelines for the Diagnosis and Management of Patients With Thoracic Aortic Disease [21]

**Table 13:** Recommendations for MR in carotid and vertebral artery disease

	Class^a^	Level^b^	Page
Carotid artery			
In patients with acute, focal ischemic neurological symptoms corresponding to the territory supplied by the left or right internal carotid artery, magnetic resonance angiography (MRA) or computed tomography angiography (CTA) is indicated to detect carotid stenosis when sonography either cannot be obtained or yields equivocal or otherwise nondiagnostic results.	I	C	15
When an extracranial source of ischemia is not identified in patients with transient retinal or hemispheric neurological symptoms of suspected ischemic origin, CTA, MRA, or selective cerebral angiography can be useful to search for intracranial vascular disease.	IIa	C	15
When the results of initial noninvasive imaging are inconclusive, additional examination by use of another imaging method is reasonable. In candidates for revascularization, MRA or CTA can be useful when results of carotid duplex ultrasonography are equivocal or indeterminate.	IIa	C	15
When intervention for significant carotid stenosis detected by carotid duplex ultrasonography is planned, MRA, CTA, or catheter-based contrast angiography can be useful to evaluate the severity of stenosis and to identify intrathoracic or intracranial vascular lesions that are not adequately assessed by duplex ultrasonography.	IIa	C	15
MRA without contrast is reasonable to assess the extent of disease in patients with symptomatic carotid atherosclerosis and renal insufficiency or extensive vascular calcification.	IIa	C	15
When complete carotid arterial occlusion is suggested by duplex ultrasonography, MRA, or CTA in patients with retinal or hemispheric neurological symptoms of suspected ischemic origin, catheter-based contrast angiography may be considered to determine whether the arterial lumen is sufficiently patent to permit carotid revascularization.	IIb	C	15
Vertebral artery			
Noninvasive imaging by CTA or MRA for detection of vertebral artery disease should be part of the initial evaluation of patients with neurological symptoms referable to the posterior circulation and those with subclavian steal syndrome.	I	C	47
In patients whose symptoms suggest posterior cerebral or cerebellar ischemia, MRA or CTA is recommended rather than ultrasound imaging for evaluation of the vertebral arteries.	I	C	47
Contrast-enhanced CTA, MRA, and catheter-based contrast angiography are useful for diagnosis of cervical artery dissection.	I	C	52

^a^Class of recommendation

^b^Level of evidence

The guideline includes a chapter explaining the principles, strength and limitations of CMR to assess the thoracic aorta (page 17). CMR “has been shown to be very accurate in the diagnosis of thoracic aortic disease, with sensitivities and specificities that are equivalent to or may exceed those for CT and TEE” (page 17). “Advantages of CMR include the ability to identify anatomic variants of aortic dissection (intramural hematoma, penetrating aortic ulceration), assess branch artery involvement, and diagnose aortic valve pathology and left ventricular dysfunction without exposing the patient to either radiation or iodinated contrast” (page 17). Throughout the text, the use of CMR in different clinical scenarios and pathologies is described and evaluated. CMR is an integral part of a number of diagnostic pathways illustrated in various figures, e.g., the aortic dissection pathway (figure 25 in the guideline, page 45) and the ascending aortic aneurysm pathway (figure 31 in the guideline, page 56). In addition, CMR is recommended for surveillance of stable and moderate thoracic aortic aneurysms (page 76), as well as for follow-up of aortic pathologies after repair or treatment (page 77, table 17 in the guideline). Finally, there are a number of specific recommendations on the use of CMR in patients with thoracic aortic disease (Table 14).

- 2010 ACCF/AHA Guideline for Assessment of Cardiovascular Risk in Asymptomatic Adults [18]

**Table 14:** Recommendations for CMR in thoracic aortic disease

	Class^a^	Level^b^	Page
Recommendations for acute thoracic aortic disease			
Urgent and definitive imaging of the aorta using transesophageal echocardiogram, computed tomographic imaging, or magnetic resonance imaging is recommended to identify or exclude thoracic aortic dissection in patients at high risk for the disease by initial screening.	I	B	43
Recommendations for Takayasu arteritis and giant cell arteritis			
The initial evaluation of Takayasu arteritis or giant cell arteritis should include thoracic aorta and branch vessel computed tomographic imaging or magnetic resonance imaging to inves- tigate the possibility of aneurysm or occlusive disease in these vessels.	I	C	28
Recommendations for aortic arch aneurysms			
For patients with isolated aortic arch aneurysms less than 4.0 cm in diameter, it is reasonable to reimage using computed tomographic imaging or magnetic resonance imaging, at 12- month intervals, to detect enlargement of the aneurysm.	IIa	C	58
For patients with isolated aortic arch aneurysms 4.0 cm or greater in diameter, it is reasonable to reimage using computed tomographic imaging or magnetic resonance imaging, at 6-month intervals, to detect enlargement of the aneurysm.	IIa	C	58
Recommendations for chronic aortic diseases in pregnancy			
For imaging of pregnant women with aortic arch, descending, or abdominal aortic dilatation, magnetic resonance imaging (without gadolinium) is recommended over computed tomographic imaging to avoid exposing both the mother and fetus to ionizing radiation. Transesophageal echocardiogram is an option for imaging of the thoracic aorta.	I	C	64
Recommendations for surveillance of thoracic aortic disease or previously repaired patients			
Computed tomographic imaging or magnetic resonance imaging of the thoracic aorta is reasonable after a Type A or B aortic dissection or after prophylactic repair of the aortic root/ ascending aorta.	IIa	C	76
Computed tomographic imaging or magnetic resonance imaging of the aorta is reasonable at 1, 3, 6, and 12 months postdissection and, if stable, annually thereafter so that any threatening enlargement can be detected in a timely fashion.	IIa	C	76
If a thoracic aortic aneurysm is only moderate in size and remains relatively stable over time, magnetic resonance imaging instead of computed tomographic imaging is reasonable to minimize the patient’s radiation exposure.	IIa	C	76

^a^Class of recommendation

^b^Level of evidence

The use of CMR for the assessment of arterial stiffness by quantifying pulse wave velocity is mentioned. However, CMR “is more costly and therefore is typically not used for testing in asymptomatic persons” (page 23). CMR is described in detail as a method for “detection and quantification of atherosclerosis. ... Examination of plaque under different contrast weighting ... allows characterization of individual plaque components, including lipid-rich necrotic core, fibrous cap status, hemorrhage, and calcification. ... It is recommended that additional large-scale multicenter trials be conducted to evaluate the possibility of using CMR in the detection of atherosclerosis in asymptomatic patients” (page 32). Yet, despite the potential of CMR for atherosclerotic plaque characterization, it is currently not recommended for screening asymptomatic subjects (Table 15).

- 2008 ACC/AHA Guidelines for the Management of Adults With Congenital Heart Disease (ACHD) [19]

**Table 15:** Recommendations for CMR for assessment of cardiovascular risk in asymptomatic adults

Recommendation for imaging of plaque	Class^a^	Level^b^	Page
MRI for detection of vascular plaque is not recommended for cardiovascular risk assessment in asymptomatic adults.	III	C	32

^a^ *Class of recommendation*

^b^ *Level of evidence*

In general, “cardiac MRI to assess ventricular anatomy and function, dimensions, myocardial perfusion, and ischemia in adults with unoperated or operated CHD is regarded as helpful” (page 29). CMR is also recommended as an integral part of regional ACHD centers (page 11, table 2 in the guideline). The guideline contains numerous text passages regarding the use of CMR in specific congenital heart diseases and clinical scenarios, such as ventricular septal defect (page 37), atrial septal defect (page 40), supravalvular aortic stenosis (page 51), and right ventricular outflow tract obstruction (page 56). To provide an example, the recommendations regarding aortic coarctation state that “MRI … with 3-dimensional reconstruction identifies the precise location and anatomy of the coarctation and entire aorta, as well as collateral vessels. … Magnetic resonance angiography may also be useful to quantify collateral flow.” (page 53). In addition, a number of specific recommendations are made (Table 16).

- 2006 ACC/AHA/ESC Guidelines for Management of Patients With Ventricular Arrhythmias and the Prevention of Sudden Cardiac Death [35]

**Table 16:** Recommendations for CMR for management of adults with congenital heart disease

	Class^a^	Level^b^	Page
Recommendations for adults with congenital heart disease (ACHD)			
Diagnostic and interventional procedures, including imaging (ie, echocardiography, MRI, or CT, advanced cardiac catheterization, and electrophysiology procedures for adults with complex and moderate CHD should be performed in a regional ACHD center with appropriate experience in CHD and in a laboratory with appropriate personnel and equipment. Personnel performing such procedures should work as part of a team with expertise in the surgical and transcatheter management of patients with CHD.	I	C	12–13
Bicuspid aortic valve disease			
MRI/CT can be beneficial to add important information about the anatomy of the thoracic aorta.	IIa	C	45
MRI may be beneficial in quantifying aortic regurgitation when other data are ambiguous or borderline.	IIb	C	45
Supravalvular aortic stenosis			
TTE and/or TEE with Doppler and either MRI or CT should be performed to assess the anatomy of the LVOT, the ascending aorta, coronary artery anatomy and flow, and main and branch pulmonary artery anatomy and flow.	I	C	50
Aortic coarctation			
Every patient with coarctation (repaired or not) should have at least 1 cardiovascular MRI or CT scan for complete evaluation of the thoracic aorta and intracranial vessels.	I	B	52
Evaluation of the coarctation repair site by MRI/CT should be performed at intervals of 5 years or less, depending on the specific anatomic findings before and after repair.	I	C	53
Supravalvular, branch, and peripheral pulmonary stenosis			
Patients with suspected supravalvular, branch, or peripheral pulmonary stenosis should have baseline imaging with echocardiography-Doppler plus 1 of the following: MRI angiography, CT angiography, or contrast angiography.	I	C	61
Congenital coronary anomalies of ectopic arterial origin			
CT or MRA is useful as the initial screening method in centers with expertise in such imaging.	I	B	65
Coronary arteriovenous fistula			
If a continuous murmur is present, its origin should be defined either by echocardiography, MRI, CT angiography, or cardiac catheterization.	I	C	67
Congenital heart disease and pulmonary arterial hypertension			
The evaluation of all ACHD patients with suspected pulmonary arterial hypertension should include noninvasive assessment of cardiovascular anatomy and potential shunting, as detailed below: Diagnostic cardiovascular imaging via TTE, TEE, MRI, or CT as appropriate.	I	C	70
After repaired of tetralogy of Fallot			
Patients with tetralogy of Fallot should have echocardiographic examinations and/or MRIs performed by staff with expertise in ACHD.	I	C	73
Dextro-Transposition of the great arteries			
Additional imaging with TEE, CT, or MRI, as appropriate, should be performed in a regional ACHD center to evaluate the great arteries and veins, as well as ventricular function, in patients with prior atrial baffle repair of d-TGA.	I	B	80
Periodic MRI or CT can be considered appropriate to evaluate the anatomy and hemodynamics in more detail in patients with prior arterial switch operation.	IIa	C	80
Congenitally corrected transposition of the great arteries			
Echocardiography-Doppler study and/or MRI should be performed yearly or at least every other year by staff trained in imaging complex CHD.	I	C	87
The following diagnostic evaluations are recommended for patients with congenitally corrected transposition of the great arteries: ECG, chest x-ray, echocardiography-Doppler study, MRI, exercise testing.	I	C	87
In patients with prior repair of congenitally corrected transposition of the great arteries, echocardiography-Doppler study and/or MRI should be performed yearly or at least every other year by staff trained in imaging complex CHD.	I	C	89
After Fontan Procedure			
All patients with prior Fontan type of repair should have periodic echocardiographic and/or magnetic resonance examinations performed by staff with expertise in ACHD.	I	C	97

^a^Class of recommendation

^b^Level of evidence

The guideline dedicates a paragraph to the strength of CMR “to evaluate both the structure and function of the beating heart. The excellent image resolution obtained with current techniques allows for the accurate quantification of chamber volumes, LV mass, and ventricular function. This is of particular value in patients with suspected arrhythmogenic RV cardiomyopathy (ARVC), in whom MRI provides excellent assessment of RV size, function, and regional wall motion and, importantly, may allow the detection of fatty infiltration within the RV myocardium. … Cardiac MRI increasingly is being applied and validated for the detection of ischemia (adenosine stress perfusion and dobutamine stress wall motion studies) and the detection and quantification of infarction/fibrosis, a substrate for VT.” In addition, CMR is mentioned to detect cardiac involvement in sarcoidosis (page 41), “to be helpful in assessing extent of disease and predicting sudden cardiac death” (page 48), in hypertrophic cardiomyopathy, and “for the evaluation of patients with ventricular tachycardia arising from the RV in the absence of defined abnormalities on conventional testing particularly to exclude ARVC” (page 59). Finally, the guideline contains one specific recommendation for CMR for accurate assessment of LV and RV function and evaluation of structural changes (Table 17).

**Table 17:** Recommendations for CMR for patients with ventricular arrhythmias and the prevention of sudden cardiac death

Recommendations for CMR for patients with ventricular arrhythmias and the prevention of sudden cardiac death	Class^a^	Level^b^	Page
MRI, cardiac computed tomography (CT), or radionuclide angiography can be useful in patients with ventricular arrhythmias when echocardiography does not provide accurate assessment of LV and RV function and/or evaluation of structural changes.	IIa	B	19

^a^Class of recommendation

^b^Level of evidence

## Discussion

This study demonstrates that CMR is mentioned in the majority of the AHA/ACC guidelines (66.7%) and that 50% of the AHA/ACC guidelines contain a total of 65 specific recommendations for when and how to use CMR. When analyzing the AHA/ACC guidelines in detail, some interesting aspects arise:

First, a look at the specific recommendations reveals that the indication category with the largest number of specific recommendations is vascular imaging. This is probably related to the fact that vascular imaging (MR angiography) is one of the most established CMR techniques. It is also worth noting that there is significant heterogeneity across the guidelines regarding whether a particular topic is only mentioned in the text, versus being included as a specific recommendation and assigned a class of recommendation and a level of evidence. Hence, to simply correlate the absolute number of specific recommendations with the importance of CMR across guideline documents may be flawed. Nevertheless, the large number of specific recommendations in the vascular disease category certainly underlines the well-defined role of CMR and MR angiography. This has also been reflected in the ESC guideline analysis, which showed in total 17 specific recommendations in the vascular category [3].

The category with the second highest number of recommendations is congenital heart disease. Patients with congenital heart disease are monitored and treated mostly in dedicated centers. The prevalence in CMR in AHA/ACC guidelines for congenital heart disease underscores the fact that CMR is commonly utilized in this population both in the preoperative state and during patient follow-up.

Regarding CMR myocardial stress testing, only eight specific recommendations exist. In relation to the number of guidelines dealing with coronary artery disease (guidelines for STEMI, NSTEMI and stable coronary artery disease), the evidence proving the diagnostic accuracy for CMR stress testing to detect coronary artery disease [2], and the dominance of coronary artery disease in clinical routine, this number suggests an underrepresentation of CMR stress testing. Conversely, CMR stress testing has a greater presence in the ESC guidelines where it is treated as equivalent with other modalities such as stress echocardiography and nuclear perfusion studies, ending up in a total of 28 specific recommendations [3]. With new data recently published, such as the CE-MARC 2 trial in 2016 that showed that CMR stress testing contributes to a lower rate of “unnecessary” invasive coronary angiographies [2], the role of CMR stress testing may increase in future editions of the AHA/ACC guidelines. The limited representation of CMR to assess viability to guide revascularization with only 3 specific recommendations can be interpreted in a similar sense.

Finally, there are only 6 specific recommendations regarding cardiomyopathies, all of which are part of the hypertrophic cardiomyopathy guidelines – a similar pattern as in the ESC guidelines [3]. In contrary, the evaluation of patients with known or suspected cardiomyopathy is the second largest indication group in clinical CMR in Europe, as expressed in the EuroCMR registry that included more than 27,000 patients [1]. This discrepancy may be attributed to the lack of a guideline covering cardiomyopathies in general. In case such a paper would be generated, there may be additional recommendations in favor of CMR, e.g., for assessment of arrhythmogenic ventricular cardiomyopathy, for risk stratification and differential diagnosis in dilated cardiomyopathy, for the diagnosis of non-compaction cardiomyopathy, and for diseases causing restrictive types of cardiomyopathies.

The use of CMR to assess myocarditis represents another discrepancy between representation in the AHA/ACC guidelines versus the level of evidence and clinical utilization in practice. Myocarditis is one of the most frequent indications for CMR in Europe [36] and the evidence for its diagnostic benefit is proven [37, 38]. In contrast, there is no single specific recommendation for CMR to assess myocarditis in the AHA/ACC guidelines. Only once in the context of differentiating acute coronary syndrome is CMR to assess myocarditis mentioned [26]. This might be attributed to the lack of a focused guideline regarding inflammatory heart diseases. In this regard, the AHA/ACC guidelines resemble the ESC guidelines; only one ESC guideline (concerning patients with ventricular arrhythmias) contains a single specific recommendation to perform CMR for risk stratification in inflammatory heart disease [39].

When analyzing those guidelines that cover overlapping topics (e.g., ischemic heart disease), there exists considerable heterogeneity in how they deal with similar topics. For example, the 2014 guideline on stable coronary artery disease contains several specific recommendations for CMR stress testing. On the other hand, similar chapters in the guideline on NSTEMI or assessment of ventricular arrhythmias contain no specific recommendations for stress CMR. This may reflect the different publication dates, as the clinical evidence and utilization of stress CMR have continued to grow. In addition, a high degree of coordination is required to synchronize recommendations for indications that appear in multiple guidelines.

When looking at those guidelines that do not mention CMR at all, it seems understandable that the guidelines dealing with “supraventricular tachycardia”, “overweight”, “lifestyle management” and “blood cholesterol” would not include recommendations for CMR. In contrast it is rather surprising - based on the topic and the common indications for CMR - that the AHA/ACC guidelines dealing with “STEMI”, “assessment of cardiovascular risk”, “secondary prevention for patients with coronary vascular disease” and “CABG” do not mention CMR at all. In comparison, the ESC-STEMI guideline from 2012 contains 2 specific recommendations (for assessment of infarct size and resting LV function; for ischemia and viability).

Four AHA/ACC guidelines mention CMR in the text without including specific recommendations (“before noncardiac surgery”, “atrial fibrillation”, “percutanoues coronary intervention”, “device therapy”). In future editions, some text passages may be accompanied by specific recommendations. On the other hand, the atrial fibrillation guideline from 2014 mentions late enhancement imaging of atrial fibrosis to predict therapeutic success [27]. Even though there is growing evidence for this application [40], its use within the CMR society is actually restricted to a small group. Thus, despite the intention to place CMR in the guidelines where the evidence supports this, modesty should also be part of the strategy to avoid the dilemma of creating non-accomplishable expectations.

### Limitations of the study

This summary is not intended to provide a comparison of the various imaging modalities in the AHA/ACC guidelines, but is rather aimed at only describing the role of CMR. Its character is more descriptive than analytical, and its layout designed to allow the reader to easily locate CMR recommendations in the guidelines, rather than to serve as a scientific meta-analysis. Additional analyses, e.g., focusing on myocardial stress testing in all guidelines regarding all available methods, will be subject of future studies. Furthermore, there are guidelines, where every detail is represented in a specific recommendation, and others, where only general recommendations are made; this leads to significant heterogeneity in the number of recommendations included in different guidelines. Naturally, the composition of the writing groups influences the content of the guidelines, and therefore the representation of CMR. However, no systematic data are available regarding the inclusion of CMR experts in the writing groups and therefore this potential influencing factor cannot be evaluated. Another factor influencing the representation of CMR in the guidelines could be the length of time it takes to generate guideline documents. It can be a 5-year process from the time of conception to publication and thus some aspects may be out of date by the time of publication. Finally, a systematic and scientific comparison of the AHA/ACC and the ESC guidelines is difficult, as most of the corresponding guidelines were not published at the same time, and many topics are not covered by guidelines of both organizations. Hence, the comparative assessments that are included must be viewed with this limitation in mind.

## Conclusions

CMR is represented in two thirds of the AHA/ACC guidelines, and these guidelines contain many recommendations in favor of the use of CMR in specific scenarios. In general, the representation of CMR is heterogeneous throughout the guidelines, with some topics (such as CMR in vascular disease and congenital heart disease) containing numerous recommendations for CMR, and others (such as those dealing with coronary artery disease) including few recommendations relative to the broad topic-related evidence. Although a direct comparison of the AHA/ACC to the ESC guidelines is difficult due to heterogeneous characteristics of both sets, CMR appears to be less represented in the AHA/ACC guidelines, in particular in coronary artery disease.

## Abbreviations

ACC: American College of Cardiology
ACHD: Adults with congenital heart disease
AHA: American Heart Association
CMR: Cardiovascular magnetic resonance
CT: Computed tomography
EF: Ejection fraction
HCM: Hypertrophic cardiomyopathy
ICD: Implantable cardioverter defibrillator
LGE: Late gadolinium enhancement
LV: Left ventricle/left ventricular
LVOT: Left ventricular outflow tract
NSTEMI: Non-ST-elevation myocardial infarction
RV: Right ventricle/right ventricular
SCD: Sudden cardiac death
STEMI: ST-elevation myocardial infarction
TEE: Transesophageal echocardiography
TTE: Transthoracic echocardiography
VT: Ventricular tachycardia
